# Unraveling the landscape of m6A RNA methylation in wound healing and scars

**DOI:** 10.1038/s41420-024-02222-w

**Published:** 2024-10-29

**Authors:** Qi Zhang, Liming Dong, Song Gong, Ting Wang

**Affiliations:** 1 Department of Plastic and Cosmetic Surgery, Tongji Hospital, Tongji Medical College, Huazhong University of Science and Technology, Wuhan, Hubei China; 2 Division of Trauma Surgery, Emergency Surgery & Surgical Critical, Tongji Trauma Center, Wuhan, China; 3 Department of Emergency and Critical Care Medicine, Tongji Hospital, Tongji Medical College, Huazhong University of Science and Technology, Wuhan, China; 4 Division of Endocrinology, Tongji Hospital, Huazhong University of Science and Technology, Wuhan, Hubei Province People’s Republic of China; 5 Department of Medical Ultrasound of Tongji Hospital of Tongji Medical College, Huazhong University of Science and Technology, Wuhan, China

**Keywords:** RNA, RNA splicing

## Abstract

Wound healing is a complex process involving sequential stages of hemostasis, inflammation, proliferation, and remodeling. Multiple cell types and factors, including underlying conditions like diabetes and bacterial colonization, can influence healing outcomes and scar formation. N6-methyladenosine (m6A), a predominant RNA modification, plays crucial roles in gene expression regulation, impacting various biological processes and diseases. m6A regulates embryonic skin morphogenesis, wound repair, and pathophysiological processes like inflammation and angiogenesis. Recent studies have highlighted the role of m6A in wound healing, scar formation, and tissue remodeling. Additionally, m6A presents a unique expression pattern in pathological wounds and scars, potentially influencing wound healing and scar formation through modulating gene expression and cellular signaling, thereby serving as potential biomarkers or therapeutic targets. Targeting m6A modifications are potential strategies to enhance wound healing and reduce scar formation. This review aims to explore the roles and mechanisms of m6A RNA methylation in wound healing and scars, and discuss current challenges and perspectives. Continued research in this field will provide significant value for optimal wound repair and scar treatment.

## Facts

- m6A RNA methylation plays a crucial role in regulating gene expression and cellular dynamics during the wound healing process, impacting key phases such as inflammation, proliferation, and remodeling.
- Proteins like METTL3, METTL14, ALKBH5, and FTO are central to the m6A modification process, influencing mRNA stability and translation, which are vital for effective tissue repair and regeneration.
- Aberrant m6A methylation patterns contribute to scar formation, with hypermethylation linked to excessive fibroblast proliferation and collagen deposition in hypertrophic scars and keloids.
- Targeting m6A modifiers presents a promising therapeutic strategy for optimizing wound healing and reducing scar formation, offering potential interventions for chronic wounds and fibrotic diseases.

## Open Questions

- What are the roles of m6A modifications on specific cell types, such as immune and endothelial cells, during wound healing, and how can these insights be harnessed for therapeutic purposes?
- How do m6A modifications on lncRNAs influence their function in wound healing, and what potential do they hold for novel therapeutic targets?
- How do systemic diseases like diabetes and autoimmune disorders affect m6A methylation processes in wound healing, and what implications does this have for treatment strategies?
- What are the long-term effects and safety considerations of m6A-targeted therapies in clinical settings for wound healing and scar management, and how can these therapies be optimized for patient-specific needs?

## Introduction

Wound healing is a complex and sophisticated physiological process to restore the integrity and functionality of wound sites [1, 2]. Usually, wound healing proceeds through the phases of four sequentially distinct and overlapping stages, including hemostasis, inflammation, proliferation, and remodeling, each crucial for the restoration of tissue regeneration [3]. Numerous cell types are instrumental in the process of wound healing, including fibroblasts, keratinocytes, endothelial cells, and immune cells [4, 5]. Underlying conditions such as diabetes, vascular diseases, lifestyle factors, and genetic predispositions that can lead to localized ischemia, can contribute to wound non-healing or exacerbation [6, 7]. The situation is further complicated by bacterial colonization and biofilm formation, obstructing the normal healing process [8]. Therefore, correcting the imbalance of cellular function and optimizing the repair phase is crucial for accelerating wound healing [9].

**Fig. 1:**
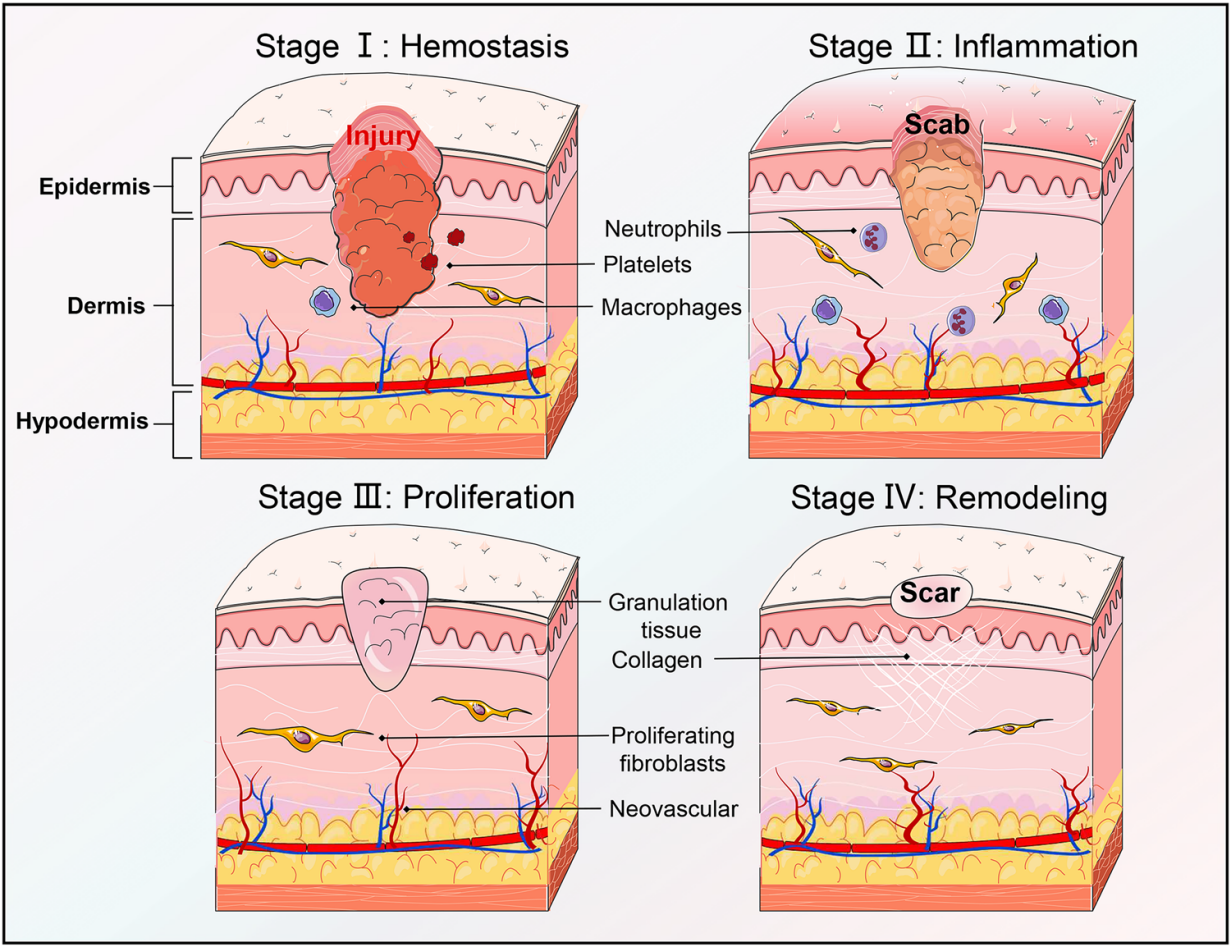
The process of wound healing and scar. Wound healing is an intricate physiological process for restoring the integrity and functionality of injured tissues. Wound healing is characterized by four sequential and overlapping phases: hemostasis, inflammation, proliferation, and remodeling. Each phase plays a pivotal role in tissue regeneration and involves the orchestration of various cell types, including fibroblasts, keratinocytes, endothelial cells, and immune cells. In the context of wound healing, certain factors like infection, hyperglycemia, and autoimmune diseases can delay the healing process, potentially leading to scar formation.

During the wound healing process, factors such as infection, hyperglycemia, and autoimmune diseases, can delay final wound healing, even potentially leading to scar formation [10–12]. The etiology of scar formation is multifaceted, encompassing genetic factors, wound size and depth, wound location, individual age and gender, and the inflammatory response during wound healing [13]. Particularly, the disruption in the balance of wound healing, such as an imbalance in collagen synthesis and degradation, can result in scar formation. The inheritance and gene regulations are important in wound healing and keloid formation [14, 15] (Fig. 1).

N6-methyladenosine (m6A), a methylation modification occurring on the 6th nitrogen atom of the adenine molecule in RNA, is the predominant form of RNA modification in mammalian cells [16]. m6A modification represents over 60% of all RNA modifications, with m6A being exceptionally abundant in mRNA and lncRNAs in higher organisms [17]. The m6A modification process is highly dynamic and reversible, and is involved multiple key enzymes, including methyltransferases “Writers”, demethylases “Erasers”, and methylation recognition proteins “Readers” [18]. These enzymes interact dynamically to maintain the cellular homeostasis of m6A levels. The regulatory mechanisms of m6A are diverse, potentially facilitating miRNA maturation, mediating alternative splicing of mRNA precursors, guiding mRNA degradation, and driving translation [19]. Additionally, m6A may induce nuclear export and X-chromosome inactivation [20]. As m6A is so crucial in gene expression regulation, the abnormalities in the m6A-related enzymes can lead to a myriad of diseases, such as cancer, autoimmune disorders, metabolic diseases, and skin diseases.

Existing studies have well-documented that m6A modifications are crucial event in embryonic skin morphogenesis, wound repair, and various pathophysiological processes, such as inflammation, angiogenesis, and fibrosis. m6A modifications of mRNA, specifically involving type XVII collagen (Col17a1), integrin β4 (Itgβ4), and α6 (Itgα6), significantly impact the expression of these proteins and the self-renewal of epidermal cells [21]. In this process, methyltransferase-like 14 (METTL14) is a key m6A methyltransferase in maintaining epidermal homeostasis. Notably, Shen et al. identified a distinct m6A methylation pattern in diabetic wound tissues, with an upregulation of the m6A eraser AlkB homolog 5 (ALKBH5) and a downregulation of m6A writers METTL3, METTL14, and Wilms tumor 1-associated protein (WTAP), indicating a disrupted m6A landscape in diabetic wound healing [22]. Genome-wide m6A profiling revealed significant alterations with differentially expressed m6A species, suggesting that m6A modifications are crucial participators in altering gene expression associated with impaired healing. Besides, m6A is linked to enhanced translation of morphogenetic signaling pathways in mouse skin embryogenesis [23]. m6A depletion disrupts cellular fate and tissue structure, with a notable downregulation in signaling and translation pathways. Upon m6A removal, highly modified mRNAs involved in RNA metabolism were increased, suggesting the roles of m6A in both translation enhancement and mRNA destabilization for rescue responses.

The above studies confirm the roles of m6A modifications in critically regulating the pathophysiological processes. m6A presents a unique expression pattern in pathological wounds and scars, potentially influencing wound healing and scar formation through modulating gene expression and cellular signaling, thereby serving as potential biomarkers or therapeutic targets. Therefore, in this review, we aim to unravel the roles and mechanisms of m6A RNA methylation in wound healing and scars, as well as the current challenges and perspectives. Continued research in this field will provide significant value for optimal wound repair and scar treatment.

## The mechanisms of m6A in gene regulation

The m6A modifications are controlled by methyltransferase, demethylases, and binding proteins, which are responsible for transferring, removing the methylation groups, and recognizing m6A adenosine bases [24, 25]. The regulations of m6A modifications affect RNA splicing, export, decay, translation, Homologous recombination-mediated DNA damage repair, and other processes [26, 27].

### m6A writers

m6A writers, a group of critical catalytic enzymes, facilitate m6A methylation modification on mRNA bases. m6A writers mainly include METTL3, METTL14, WTAP, and KIAA1492, along with other regulatory subunits, such as RNA-binding motif protein 15 (RBM15), RBM15B, Vir-like m6A methyltransferase associated (VIRMA, also known as KIAA1429 or Virilizer), Cbl proto-oncogene like 1 (CBLL1, also known as Hakai), and zinc finger CCCH-type containing 13 (ZC3H13) [28, 29]. These proteins form a multicomponent m6A methyltransferase complex (MTC) to execute their catalytic function [30]. METTL3 and METTL14 possess key catalytic structural domains and form a heterodimer complex [31]. METTL3 serves as the catalytically active subunit, while METTL14 plays a crucial role in substrate recognition [32]. This METTL3-METTL14 complex mediates m6A deposition on mammalian mRNAs, and the knockdown of either protein substantially reduces m6A mRNA levels [33]. WTAP, although lacking a conserved catalytic methylation domain, is instrumental in coordinating the localization of the METTL3-METTL14 heterodimer into nuclear speckles [34]. This coordination facilitates m6A deposition at selective transcripts and regions, thereby regulating the recruitment of the m6A MTC to mRNA targets. In addition to the MTC, certain m6A methyltransferases, such as METTL16, METTL5, and zinc finger ZCCHC4, directly catalyze m6A modification in RNA molecules [35, 36]. In summary, m6A writers, including the MTC and other methyltransferases, play a pivotal role in mRNA methylation, thereby influencing various biological processes and diseases.

### m6A erasers

The m6A erasers mainly include fat-mass and obesity-associated protein (FTO), ALKBH5, ALKBH3, and ALKBH1 [37–39]. These proteins can selectively reverse m6A to adenosine within nuclear RNA. FTO was first identified as an m6A demethylase in 2011, and possesses the capability to remove the methyl group from m6A and N6,2-O-dimethyladenosine (m6Am) in RNA, the latter primarily found in the 5’-UTR [40, 41]. Unique to FTO within the AlkB family is a distinct long loop structure at the C-terminus, enabling demethylation of methylated single-stranded DNA or RNA. ALKBH5, the second m6A demethylase discovered in 2013, is primarily localized in the nucleus [42]. ALKBH5 can remove the m6A modification from nuclear RNA, primarily mRNA, impacting mRNA export, splicing, and stability [43]. ALKBH5 has a unique coiled-coil structure at the N-terminus [44]. In essence, m6A erasers such as FTO and ALKBH5, play pivotal roles in RNA modification, with their unique demethylase activity influencing various biological processes and diseases [45].

### m6A readers

**Fig. 2:**
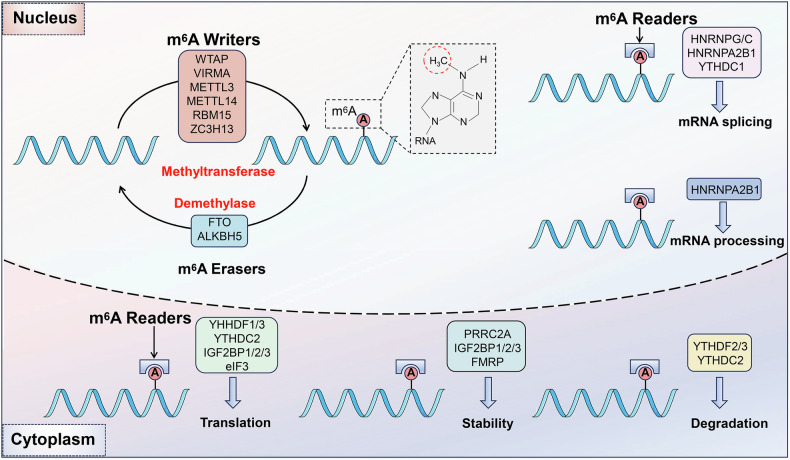
Molecular mechanism of m6A methylation. m6A methylation is governed by methyltransferases “writers”, demethylases “erasers”, and binding proteins “readers”. Writers, including METTL3, METTL14, and WTAP, form a multicomponent methyltransferase complex (MTC) that catalyzes m6A methylation. METTL3 and METTL14, as the core of the MTC, play essential roles in catalysis and substrate recognition, respectively. Erasers, such as FTO and ALKBH5, are part of the α-ketoglutarate-dependent dioxygenase family that reverses m6A to adenosine, impacting mRNA export, splicing, and stability. Readers, including YTHDF1-3, recognize and bind to m6A modifications, influencing mRNA processes like translation, stabilization, and splicing. These three classes of proteins collectively regulate m6A modifications, affecting various biological processes and diseases.

m6A readers are proteins that identify and bind to m6A modifications, thus influencing specific biological functions of modified mRNA [46]. These include YT521-Bhomology (YTH) domain-containing proteins, such as YTHDF1, YTHDC2, and YTHDC3 [47, 48]. These proteins participate in various mRNA processes, including translation, stabilization, splicing, and nuclear export. YTHDF1-3, has a YTH domain at the C-terminus, facilitating RNA-specific binding and recognizing m6A-modified mRNA in the cytoplasm, while YTHDC1-2 primarily functions in the nucleus [49]. YTHDF proteins, which are ubiquitously present, bind to m6A-modified RNA to influence RNA transcription and processing [50]. YTHDC proteins, highly expressed in the nervous system, bind to m6A-modified RNA and regulate it in a modification-specific manner. They can also affect RNA stability and degradation processes. In summary, m6A readers are crucial determinants in recognizing and interacting with m6A-modified mRNA (Fig. 2).

## Wound healing

### Cellular mechanisms of m6A modification in wound healing

METTL3 is a crucial regulator of naïve pluripotency termination in murine embryonic stem cells, while its knockout results in m6A depletion in mRNAs, impaired state transition, and early embryonic lethality [51, 52]. METTL3 could modulate heterochromatin in mouse embryonic stem cells by interacting with YTHDC1, SETDB1, and TRIM28, highlighting its critical role in chromatin regulation and mammalian development [51]. The differentiation and paracrine capability of adipose-derived mesenchymal stem cells (ADSCs) and bone marrow-derived mesenchymal stem cells (BMSCs) could be regulated by regulating METTL3 [53, 54]. Zhou et al. illustrated the pivotal role of m6A modification in ADSCs, particularly in enhancing lymphangiogenesis and wound healing in diabetic foot ulcers (DFUs) [55]. Through METTL3-mediated m6A modification of VEGF-C, ADSCs could promote VEGFR3-mediated lymphangiogenesis, for improving DFU wound healing. Therefore, m6A modification was a critical regulatory mechanism in ADSC-mediated skin healing.

As the most common demethylases, ALKBH5 and FTO have been reported to be involved in wound healing. Zan et al. reported that demethylase ALKBH5 was specifically upregulated at the wound edge [56]. ALKBH5 served as an accelerator for wound re-epithelialization by removing m6A modification from PELI2 mRNA, thereby stabilizing it in a YTHDF2-dependent manner by integrated high-throughput analysis. The absence of ALKBH5 in Alkbh5–/– mice led to reduced keratinocyte migration and delayed wound healing. The addition of exogenous PELI2 could partially rescue the impaired migration and re-epithelialization in ALKBH5-deficient cells. Collectively, ALKBH5 accelerated wound re-epithelialization, presenting a potential target for developing m6A-based therapies aimed at treating refractory wounds. Wang et al. found that demethylase FTO modulated burn wound healing through m6A methylation modification by inhibiting TFPI-2, thereby enhancing the proliferation, migration, and angiogenesis of heat-stimulated keratinocytes [57]. The interplay between FTO and tissue factor pathway inhibitor-2 (TFPI-2) not only accelerated wound healing, but also mitigated depressive-like behaviors in a burn injury model. Consequently, FTO-mediated demethylation of TFPI-2, emerged as a promising therapeutic target for improving both physical healing and psychological outcomes following burn injuries.

YTHDC1 could modulate the inflammation response in human keratinocytes by regulating miR-146a/TRAF6/IRAK1 Axis [58]. The downregulation of the m6A reading protein YTHDC1 under hyperglycemic stress was associated with decreased levels of the important autophagy receptor SQSTM1/p62, which affected autophagy flux and wound healing in keratinocytes [59]. Additionally, YTHDC1 was essential for maintaining the stability of SQSTM1 mRNA, with its knockdown leading to enhanced mRNA degradation and disrupted autophagic processes. m6A modifications perform a coordinated regulatory role in the dynamics of autophagy in diabetic keratinocytes and demonstrate the potential for diabetic wound healing.

### Epigenetic regulation of m6A modification in wound healing

Non-coding RNAs (ncRNAs) are a diverse group of RNA transcripts that do not encode proteins, including microRNAs (miRNAs), long non-coding RNAs (lncRNAs), circular RNAs (circRNAs), small interfering RNAs (siRNAs), and piwi-interacting RNAs (piRNAs) [60, 61]. ncRNAs play critical roles in regulating gene expression and are involved in the epigenetic mechanisms contributing to various disease pathogenesis [62, 63]. The expression and abundance of ncRNAs and m6A-modified genes undergo significant changes post-injury in skin tissues [64, 65]. These ncRNAs, crucial for gene expression regulation and cellular behavior, are heavily influenced by m6A methylation [66]. m6A modification on ncRNAs is essential for modulating their stability and function, impacting key wound healing processes and potentially affecting recovery outcomes [67]. In a comprehensive m6A methylation landscape of mRNA and lncRNA transcriptomes in human skin tissue post-burn, Ran et al. identified significant hypermethylation in 65 mRNAs and 39 lncRNAs, and pronounced hypomethylation in 5492 mRNAs and 754 lncRNAs, with the latter group showing suppressed expression that inhibited numerous wound healing processes and pathways [68]. Furthermore, there was a significant downregulation of m6A regulatory factors, including METTL14, METTL16, ALKBH5, fragile X mental retardation 1 (FMR1), and heterogeneous nuclear ribonucleoprotein C (HNRNPC), in post-burn tissues. These m6A modifications might be a potential mechanism affecting wound infection and healing.

m6A modification has been confirmed as a pivotal epitranscriptomic mechanism that regulates the stemness of epidermal progenitor cells, essential for skin rejuvenation and wound healing [69]. m6A modification of the lncRNA Pvt1 was crucial for maintaining the self-renewal capacity of epidermal progenitor cells. Disruption of m6A methylation, either through methyltransferase deletion or Pvt1 ablation, significantly impaired skin healing processes. In terms of the mechanism, m6A modification was related to enhanced Pvt1 interaction with MYC within epidermal progenitor cells. In diabetic wound healing, the m6A modification of lncCCKAR5 enhanced its interaction with MKRN2 and LMNA, promoting autophagy and inhibiting apoptosis in human umbilical cord mesenchymal stem cells (hUCMSCs) under high glucose conditions [70]. m6A-modified lncCCKAR5 served as an essential platform, orchestrating the ubiquitin-dependent breakdown of LMNA, which was integral to the cytoskeletal function and autophagy process. This finding highlighted the m6A role in the lncCCKAR5/LMNA/MKRN2 complex, offering a novel therapeutic target for improving diabetic wound healing facilitated via hUCMSCs.

### m6A in inflammatory response and healing

Insulin-like growth factor 2 mRNA-binding proteins (IGF2BP), including IGF2BP1, IGF2BP2, and IGF2BP3, are oncofetal RNA-binding proteins that play crucial roles in cell function and biological functions, particularly in processes such as cell polarization, migration, and proliferation [71]. These proteins, which are predominantly expressed during embryogenesis and re-expressed in aggressive cancers and dysregulated diseases, interact with mRNAs in cytoplasmic ribonucleoprotein complexes [16, 72].

**Fig. 3:**
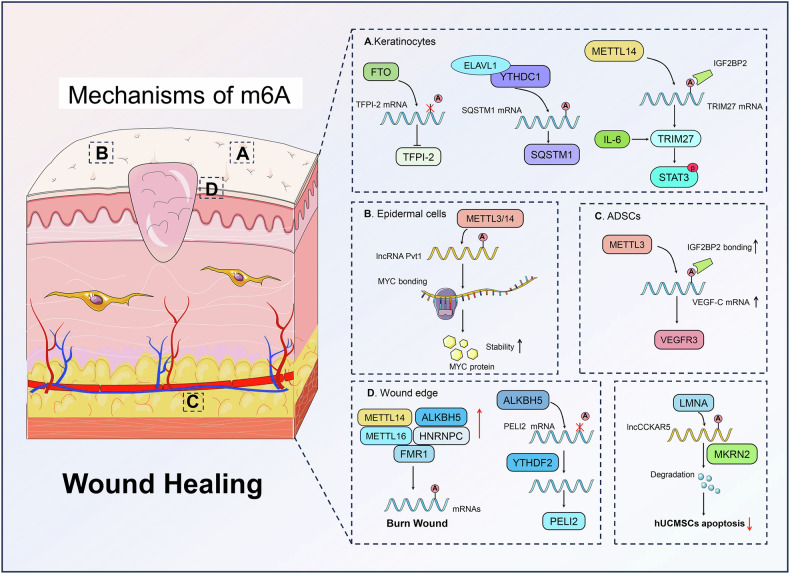
The roles of m6A methylation in regulating wound healing. m6A methylation is critical in wound healing, and involves key players including METTL3, METTL14, ALKBH5, FTO, and YTHDC1. m6A writer METTL3 modulates stem cell differentiation and tissue regeneration, while METTL14 is involved in the epigenetic regulation of ncRNAs. ALKBH5 and FTO, as m6A erasers, accelerate wound healing by influencing mRNA stability and keratinocyte function. YTHDC1, an m6A reader, regulates inflammation and autophagy in keratinocytes, crucial for diabetic wound healing. Together, these m6A modifiers serve as potential therapeutic targets for wound healing and related skin diseases, emphasizing the significance of m6A modification in cellular and epigenetic regulation of wound healing processes.

IGF2BP2 is related to aberrant keratinocyte differentiation. IGF2BP2 could be targeted by the microRNA let-7b to regulate keratinocyte migration, thereby significantly delaying re-epithelialization during skin wound healing [73]. IGF2BP2 was also identified as a critical mediator in epidermal keratinocyte differentiation, where it partnered with lnc-DC to stabilize ZNF750 mRNA, thereby enhancing the expression of downstream genes TINCR and KLF4 under the regulatory influence of GRHL3 [74]. In addition, Chen et al. unveiled that the m6A reader IGF2BP2 in keratinocyte cytopathy, where it recognized and interacted with m6A-methylated TRIM27, facilitated by the m6A writer METTL14 [75]. This METTL14/TRIM27/IGF2BP2 signaling axis was instrumental in activating the IL-6/STAT3 signaling pathway, which was implicated in various skin inflammatory conditions. The findings highlighted the potential of targeting IGF2BP2 and the m6A methylation process as a novel therapeutic approach for skin inflammatory diseases (Fig. 3).

## Scars

The morphological characteristics of scars determine their classification into flat scars, atrophic scars, hypertrophic scars, and keloids [76]. Flat scars are typically the result of normal wound healing, while atrophic scars form depressions in the skin due to collagen loss or underlying tissue damage [77]. In contrast, both hypertrophic scars and keloids result from excessive collagen accumulation [78, 79]. However, keloids extend beyond the original wound boundaries and do not naturally regress over time, distinguishing them from hypertrophic scars [80]. Current research on m6A regulation in scars primarily focuses on keloids, likely due to the relative ease of sample collection from these tissues, which facilitates the analysis of m6A expression and mechanisms.

### Scar-specific m6A modification patterns

The heterogeneity and gene modification are a key aspect in understanding scar development [81, 82]. The use of m6A sequencing technology has been instrumental in revealing the expression patterns and abundance of m6A in scar tissues [83]. These findings provide crucial insights into the dynamic regulation of m6A methylation in scars. By m6A sequencing and RNA sequencing, Liu et al. showed that there was a dynamic m6A regulation in scar tissue compared to normal skin [84]. The altered m6A modification patterns in hyperplastic scar (HS) were closely linked to fibrosis pathways, and the differentially expressed mRNA transcripts were found in HS samples with hyper-methylated or hypo-methylated m6A peaks, thus confirming the role of m6A in scar formation and fibrotic processes. Lin et al. revealed a distinct m6A methylation pattern in keloid tissues and suggested a hyper-methylated state in keloid fibroblasts [85]. This hyper-m6A-modified highly expressed Wnt/β-catenin pathway in keloid fibroblast facilitated the keloid progression.

### m6A mechanism in keloid progression

METTL3 is a key m6A methyltransferase, and can modulate mRNA translation and various biological processes, including cell cycle progression, proliferation, apoptosis, migration, invasion, differentiation, and inflammation [86]. Notably, METTL3-mediated m6A modification exacerbated arsenite-induced skin damage via JAK2/STAT3/Krt signaling [59]. In the exploration involving epidermal progenitors, deletion of METTL3 led to severe developmental disruptions, including impaired hair follicle morphogenesis and defects in cell adhesion and polarity [87]. METTL3 facilitated the m6A-mediated degradation of mRNAs that encode key histone-modifying enzymes, regulating their expression and impacting chromatin structure and gene expression [88]. The absence of METTL3 results in increased stability and expression of these mRNAs, causing extensive gene expression abnormalities and phenotypic defects in epithelial tissues, highlighting the critical role of m6A in epithelial development and self-renewal [89].

FTO exhibits a diverse range of biological functions, such as RNA metabolism, gene expression, and epigenetic modifications [90, 91]. Additionally, as a gene linked to obesity, FTO impacts bone formation by modulating the process of fat cell development. Alterations in FTO influence m6A methylation patterns on RNA, thereby modulating cellular processes such as proliferation, differentiation, and apoptosis [92, 93]. Ren et al. reported that there were aberrantly ordering and proliferation of fibroblasts in the keloid tissue, with increased expression of the m6A demethylase FTO dysregulation of RNA methylation processes in keloid pathogenesis [94]. FTO overexpression promoted fibroblast migration and upregulates collagen type I alpha 1 chain (COL1A1) and α-smooth muscle actin (α-SMA) by modulating m6A modification and mRNA stability. This highlighted the significance of FTO in keloid development, suggesting that targeting FTO-mediated m6A demethylation could offer a new therapeutic approach for keloid treatment.

IGF2BP3 is an integral component of the m6A reading machinery and mediates mRNA stability and regulates gene expression [95, 96]. The aberrant expression of IGF2BP3 is found in various cancer types and inflammatory diseases [97, 98]. Yang et al. identified a significant upregulation of the m6A gene IGF2BP3 in keloid patients, suggesting its potential role in keloid pathogenesis [99]. The m6A genes were involved in cell division, proliferation, and metabolism, as well as significant differences in immunity-related pathways.

**Fig. 4:**
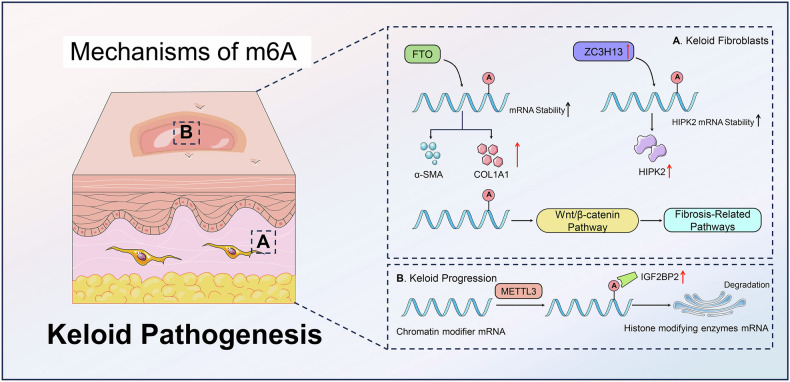
The roles of m6A methylation in regulating scars. The m6A demethylase FTO promoted fibroblast migration and COL1A1 and α-SMA expression for keloid development. IGF2BP3 was involved in keloid pathogenesis. ZC3H13 enhanced cell proliferation and migration while reducing apoptosis in human keloid fibroblasts by ZC3H13-mediated HIPK2 mRNA m6A modification. Targeting FTO, IGF2BP3, and ZC3H13 could offer a new therapeutic approach for keloid treatment.

ZC3H13 acts as a crucial component of the RNA m6A methylation regulatory complex, anchoring it within the nucleus and interacting with proteins such as WTAP and Virilizer, to modulate m6A levels on RNA [100, 101]. ZC3H13 is essential for the localization and function of the methylation complex, influencing m6A modification dynamics and playing a pivotal role in the self-renewal of mouse embryonic stem cells [102]. In the pathogenesis of keloids, the m6A regulator ZC3H13 was found to be upregulated. ZC3H13 enhanced cell proliferation and migration while reducing apoptosis in human keloid fibroblasts [103]. ZC3H13 overexpression increased m6A modification of HIPK2 mRNA, leading to heightened mRNA stability and elevated HIPK2 expression in keloid tissues. Therefore, ZC3H13-mediated HIPK2 mRNA m6A modification could promote keloid progression by the post-transcriptional regulation of HIPK2 (Fig. 4).

## Discussion

As discussed above, in the wound healing process, the expression and abundance of m6A-modified genes in skin tissues undergo significant changes. m6A modifications are critical in regulating the stability and function of genes, affecting key processes in wound healing and potentially influencing recovery outcomes. Disturbances in the m6A landscape, characterized by hyper- and hypomethylation, inhibit wound healing pathways and processes. Targeting m6A-related mechanisms may provide novel therapeutic approaches to optimize wound healing and reduce scar formation.

m6A modifications exhibit distinct expression patterns and mechanisms across various skin conditions, including wounds, scars, and other diseases, yet several commonalities also emerge [104]. In wound healing, m6A levels dynamically change at the wound edge, promoting epithelial regeneration and cell migration, essential for tissue repair. Conversely, in scar formation, particularly hypertrophic scars and keloids, hypermethylation of m6A leads to excessive fibroblast proliferation and collagen deposition. In skin cancers like melanoma and squamous cell carcinoma, aberrant m6A modifications are linked to tumorigenesis and progression, with dysregulation of regulators such as METTL3 and FTO enhancing tumor cell proliferation and invasion. In inflammatory skin diseases like psoriasis and eczema, specific m6A modification patterns influence the expression of inflammatory cytokines and immune response regulation [105–107]. Despite these differences, commonalities include the universal regulation of gene expression by m6A through modulation of mRNA stability, splicing, translation, and decay, impacting cellular functions such as differentiation, proliferation, and apoptosis. Dysregulation of m6A regulators consistently correlates with abnormal cell behavior across conditions, and m6A role in modulating inflammatory responses underscores its broad regulatory impact and potential as a therapeutic target in diverse skin diseases [108].

The important role of m6A RNA methylation in wound healing and scar formation has been widely demonstrated, but some critical research gaps remain. The first thing worth noting is the role of m6A modifications on specific cells during wound healing. The existing studies have extensively explored the m6A roles and mechanisms of keratinocytes and fibroblasts, but immune and endothelial cells have not been relatively investigated [109]. Uncovering the roles of m6A influence across these diverse cell types could excavate novel regulatory mechanisms essential for effective tissue repair [110]. Additionally, the functional implications of lncRNAs in wound healing are poorly understood despite their potential regulatory capabilities mediated by m6A [111]. This reinforces the necessity for focusing studies on lncRNAs to characterize their specific contribution to the cellular and molecular landscapes of wound repair.

Furthermore, the impact of systemic diseases, such as diabetes and autoimmune disorders, might impact the m6A methylation process in wound healing. This is very important as these disorders often complicate the healing process and may confound m6A-based targeted therapeutic strategies. Moreover, while the molecular mechanisms behind scar formation are increasingly appreciated, strategies to prevent excessive scarring are still lacking. There is a need to investigate how m6A modifications change dynamically after injury and their correlation with healing outcomes. This could lead to the development of novel interventions to prevent or reduce scar formation, thereby improving clinical outcomes for patients with chronic wounds or fibrotic diseases.

Notably, there are still some limitations and challenges of m6A in wound healing and scars. Scar formation is the final stage of wound healing but is often not observed in mouse models. Various models and methods have been used to study the mechanisms of scar formation. Large animal models such as pigs and rabbits are often employed because their structure and healing process are similar to that of human skin. Human skin graft models, in which human skin is transplanted onto immunodeficient nude mice or SCID mice, more accurately simulate human wound healing and scar formation. In vitro models, including 3D skin models and organoid culture systems, allow controlled studies of relevant molecular and cellular mechanisms. Clinical sample analysis, including collection and examination of wound and scar tissue from human patients, combined with high-throughput sequencing and other molecular biology techniques, provides valuable insight into the molecular pathways of scar formation. However, no single model can fully recapitulate the pathological characteristics of human scar formation. In this review, references related to scar and keloid formation primarily involve models using keloid patient skin tissues, normal human skin tissues, and isolated skin fibroblasts. Our review does not encompass studies involving animal models. Future research should utilize animal models to validate and extend these findings, offering a deeper understanding of scar and keloid formation.

Additionally, most studies utilize animal models or cell culture models, which may not fully simulate the complex human wound-healing microenvironment [112, 113]. Bridging these findings to clinical applications requires more human-based studies to validate the therapeutic potential of targeting m6A modifications. Then, the entire network of m6A regulatory proteins and their interactions remain incompletely characterized in the context of wound healing. A deeper understanding of this network is essential to manipulate these pathways effectively for therapeutic purposes, including the development of m6A modulators that could either enhance or inhibit methylation as needed. The dose-response relationship between m6A methylation levels and wound closure rates is another underdeveloped area [114]. Establishing this relationship could help in designing dosage guidelines for therapeutic interventions targeting m6A. Furthermore, the long-term effects of altering m6A methylation in clinical settings are not well understood, highlighting a need for longitudinal studies to assess the safety and efficacy of m6A-targeted therapies [115]. To bridge the gap between basic research and clinical application of m6A RNA methylation in wound healing and scar formation, we propose a multifaceted approach. First, developing robust preclinical models is essential to study the effects of modulating m6A methylation in these processes. Second, identifying m6A-related biomarkers that can predict wound healing outcomes and scar formation will facilitate their use in clinical settings. Third, designing and conducting clinical trials to test m6A-targeted therapies is crucial for assessing their safety, efficacy, and potential side effects in human subjects.

Looking forward, the development of new diagnostic tools utilizing m6A as biomarkers is also full of prospects. Such tools might revolutionize how clinicians monitor wound healing and manage scar formation, leading to more personalized and effective treatment strategies. Precision medicine approaches could also benefit from a better understanding of individual m6A methylation profiles, potentially leading to customized therapies that address the unique genetic and epigenetic landscape of the individualized wound healing process. Moreover, the application of synthetic biology to modulate m6A methylation presents an innovative strategy to optimize healing and minimize scarring. By designing molecules that can specifically alter m6A landscapes, researchers could directly modulate gene expression profiles critical for tissue repair. Additionally, fostering interdisciplinary research collaborations among fields, such as biomedical science, materials science, and nanotechnology, could accelerate the development of novel therapeutic strategies, ultimately transforming the management of wounds and reducing the burden of scarring in clinical practice [116, 117]. These future directions not only highlight the potential of m6A research in wound healing but also emphasize the transformative impact on regenerative medicine and dermatology.

## Conclusion

m6A modification significantly influences wound healing and scar formation through intricate regulation of gene expression and cellular dynamics. Key m6A regulatory proteins, such as METTL3, METTL14, and WTAP, orchestrate the methylation process, enhancing the stability and translation of critical transcripts like Col17a1, Itgβ4, and Itgα6, which are essential for epidermal cell self-renewal and skin morphogenesis. Meanwhile, demethylases like ALKBH5 and FTO modulate the healing process by removing m6A marks, thereby affecting mRNA decay and protein synthesis pathways involved in tissue repair and inflammation. m6A readers including YTHDF and IGF2BP2, recognize and bind m6A-modified RNAs, influencing cellular responses such as keratinocyte migration and inflammatory signaling. These interactions highlight the dynamic and reversible nature of m6A modifications, showing their potential as therapeutic targets for enhancing tissue regeneration and minimizing scar formation in wound healing and scar formation.
